# Plasma NfL is associated with the *APOE* ε4 allele, brain imaging measurements of neurodegeneration, and lower recall memory scores in cognitively unimpaired late-middle-aged and older adults

**DOI:** 10.1186/s13195-023-01221-w

**Published:** 2023-04-10

**Authors:** Michael Malek-Ahmadi, Yi Su, Valentina Ghisays, Ji Luo, Vivek Devadas, Yinghua Chen, Wendy Lee, Hillary Protas, Kewei Chen, Henrik Zetterberg, Kaj Blennow, Richard J. Caselli, Eric M. Reiman

**Affiliations:** 1 Banner Alzheimer’s Institute, 901 E. Willetta St., Phoenix, AZ 85006 USA; 2 Department of Psychiatry and Neurochemistry, Institute of Neuroscience and Physiology, The Sahlgrenska Academy, University of Gothenburg, Gothenburg, Sweden; 3 Clinical Neurochemistry Laboratory, Sahlgrenska University Hospital, Mölndal, Sweden; 4 Department of Neurodegenerative Disease, UCL Institute of Neurology, Queen Square, London, UK; 5 UK Dementia Research Institute at UCL, London, UK; 6 Hong Kong Center for Neurodegenerative Diseases, Hong Kong, China; 7 Mayo Clinic Arizona, Scottsdale, AZ USA; 8 Translation Genomics Research Institute, Phoenix, AZ USA; 9 University of Arizona, Phoenix, AZ USA; 10 Arizona State University, Tempe, AZ USA

## Abstract

**Background:**

Plasma neurofilament light (NfL) is an indicator of neurodegeneration and/or neuroaxonal injury in persons with Alzheimer’s disease (AD) and a wide range of other neurological disorders. Here, we characterized and compared plasma NfL concentrations in cognitively unimpaired (CU) late-middle-aged and older adults with two, one, or no copies of the *APOE* ε4 allele, the major genetic risk factor for AD. We then assessed plasma NfL associations with brain imaging measurements of AD-related neurodegeneration (hippocampal atrophy and a hypometabolic convergence index [HCI]), brain imaging measurements of amyloid-β plaque burden, tau tangle burden and white matter hyperintensity volume (WMHV), and delayed and total recall memory scores.

**Methods:**

Plasma NfL concentrations were measured in 543 CU 69 ± 9 year-old participants in the Arizona *APOE* Cohort Study, including 66 *APOE* ε4 homozygotes (HM), 165 heterozygotes (HT), and 312 non-carriers (NC). Robust regression models were used to characterize plasma NfL associations with *APOE* ε4 allelic dose before and after adjustment for age, sex, and education. They were also used to characterize plasma NfL associations with MRI-based hippocampal volume and WMHV measurements, an FDG PET-based HCI, mean cortical PiB PET measurements of amyloid-β plaque burden and meta-region-of-interest (meta-ROI) flortaucipir PET measurements of tau tangle burden, and Auditory Verbal Learning Test (AVLT) Delayed and Total Recall Memory scores.

**Results:**

After the adjustments noted above, plasma NfL levels were significantly greater in *APOE* ε4 homozygotes and heterozygotes than non-carriers and significantly associated with smaller hippocampal volumes (*r* =  − 0.43), greater tangle burden in the entorhinal cortex and inferior temporal lobes (*r* = 0.49, *r* = 0.52, respectively), and lower delayed (*r* =  − 0.27), and total (*r* =  − 0.27) recall memory scores (*p* < 0.001). NfL levels were not significantly associated with PET measurements of amyloid-β plaque or total tangle burden.

**Conclusions:**

Plasma NfL concentrations are associated with the *APOE* ε4 allele, brain imaging biomarkers of neurodegeneration, and less good recall memory in CU late-middle-aged and older adults, supporting its value as an indicator of neurodegeneration in the preclinical study of AD.

**Supplementary Information:**

The online version contains supplementary material available at 10.1186/s13195-023-01221-w.

## Introduction

Biomarkers can be used to detect and track the progressive amyloid-β (Aβ) plaque (A), tau tangle (T), and neurodegenerative changes (N) associated with Alzheimer’s disease (AD) pathology before the onset of clinical symptoms. While brain imaging and cerebrospinal fluid (CSF) biomarkers have been used most extensively, emerging blood-based biomarkers (BBBMs) promise to play an increasingly important role in this endeavor [1, 2]. Neurofilament light (NfL) is a cytoskeleton protein expressed only in neurons. It can be measured in brain, CSF, serum, and plasma to provide an indicator of neuronal injury and/or degeneration in a wide range of neurological disorders [3–7]. Findings from studies of autosomal dominant Alzheimer’s disease (ADAD) mutation carriers have found that plasma NfL begins to increase between 16 [8] and 22 [9] years prior to the onset of mild cognitive impairment (MCI) while cross-sectional findings have also demonstrated that higher serum NfL levels are associated with decreased white matter integrity [10].

Others report that plasma NfL correlates well with post-mortem neurofibrillary tangle load as well as immunohistochemical measurements of NfL in brain tissue [3] while others have demonstrated that increases in CSF-derived NfL are associated with decreased cerebral glucose hypometabolism [5, 6] as well as decreased cortical thickness [7] in regions that are preferentially affected by AD. In a cohort of cognitively unimpaired (CU) autosomal dominant presenilin-1 (PSEN) E280A mutation carriers, higher plasma NfL levels were associated with increased tau burden and decreased cognitive performance [8, 9, 11, 12].

Here, we capitalized on blood samples from CU late middle-aged and older adults with two, one, or no copies of the apolipoprotein E (*APOE*) ε4 allele, the major AD susceptibility gene, who have been followed in the longstanding longitudinal Arizona *APOE* Cohort Study. We sought to test the hypotheses that plasma NfL levels are associated with (a) *APOE* ε4 allelic dose, (b) FDG-PET and MRI biomarkers of neurodegeneration in brain regions preferentially affected by AD, and (c) lower recall memory test scores. We also explored associations with other MRI measurements.

## Methods

### Arizona APOE cohort

Blood samples and data for this study came from the Arizona *APOE* Cohort [13–15], a longstanding longitudinal study of cognitively unimpaired (CU) persons with two, one, and no copies of the *APOE* ε4 allele. The study used newspaper advertisements were used to recruit cognitively unimpaired volunteers throughout the adult age range between 1994 and 2017, characterized their *APOE* genotypes, and enrolled *APOE* ε4 homozygotes, heterozygotes, and non-carriers who were initially matched for their age, sex, and educational level, and have followed them every 1 to 2 years using a battery of clinical ratings, cognitive tests, a growing number of brain imaging, CSF, and emerging blood-based biomarker measures. Participants gave written and informed consent, and the study was approved by institutional review boards for Mayo Clinic and Banner Health.

Assessments included a neurological examination, the Folstein Mini-Mental Status Exam (MMSE) [16], the Hamilton Depression (Ham-D) Rating Scale [17], the Functional Activities Questionnaire (FAQ) [18], Instrumental Activities of Daily Living (IADL) [19], the Structured Psychiatric Interview for DSM-IIIR [20], and an extensive battery of neuropsychological tests and other clinical ratings [21]. None met the published criteria for amnestic mild cognitive impairment (aMCI) [22], AD [23], other forms of dementia, or major depressive disorder [20] at study entry.

**Fig. 1:**
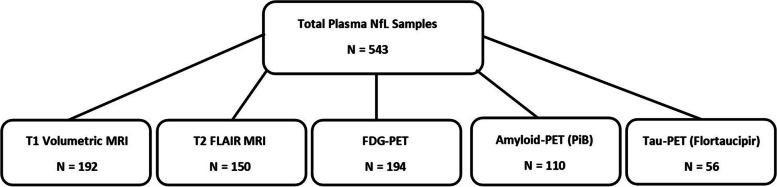
Flowchart of imaging modalities with sample sizes used in the analyses

The current analysis included 543 subjects (NC = 312, HT = 165, HM = 66) with a mean age of 68.8 ± 8.6 years (range = 50–91) and mean education level was 16.0 ± 2.2 years. Seventy-two percent of the sample was female. Since many of the neuroimaging and blood biomarker assessments were added at different times, subject visits with the highest number of available imaging and biomarker data were used. For many of the imaging modalities, the number of available subjects was smaller than the total sample size (Fig. 1) due to a variety of factors (changes in MRI acquisition procedures, scan quality issues, imaging not performed within 6 months of the neuropsychology assessment, addition of amyloid- and tau-PET measures several years after the study was first initiated).

### Plasma NfL assay

Plasma NfL measurements were performed at the Clinical Neurochemistry Laboratory, University of Gothenburg, Sweden, using the NF-Light kit on a Single Molecular Array (Simoa) HD-X Analyzer (Quanterix, Billerica, MA) according to the manufacturer’s instructions. Calibrators were run in duplicates, and obvious outlier calibrator replicates were masked before curve fitting. Samples were diluted fourfold and run in singlicates. All measurements were made without information on any clinical data. The dynamic range of the assay was 1.9–1800 pg/mL. Two QC plasma samples were run in duplicates in the beginning and the end of each run. For the QC sample with a concentration of 10.8 pg/mL, repeatability was 4.8% and intermediate precision 6.2%, while for the QC sample with a concentration of 47.7 pg/mL, repeatability was 3.3% and intermediate precision 4.6%. The plasma samples used for this assay were drawn on the same day that the imaging assessments were performed; however, there was some delay between sample collection and the neuropsychological assessments which was an average of 40.2 ± 99.8 days.

### FDG, amyloid, and tau PET

Fluorodeoxyglucose (FDG) PET which provides information about regional cerebral metabolic rates for glucose (CMRgl) has been extensively used to detect and track CMRgl declines in brain regions that are preferentially affected by AD. It has been shown by our group and others to detect and track precuneus, posterior cingulate, temporal, partial, and frontal CMRgl declines starting many years before the onset of symptoms in *APOE* ε4 homozygotes, heterozygotes, and other persons at risk for AD [24–26]. We previously developed an FDG-PET-derived hypometabolic convergence index (HCI) [27] to provide an indicator of AD-related CMRgl reductions in a single measurement, and we now use this measurement to provide an FDG PET indicator of neurodegeneration [28]. Greater HCI values indicate lower glucose metabolism in cortical regions associated with AD (*n* = 194).

PiB PET provides information about neuritic Aβ plaque deposition. PiB PET images from 110 participants were used to assess mean cortical-to-cerebellar standard uptake value ratios (SUVR) [29, 30] using the median-uptake of voxels in prefrontal, orbitofrontal, parietal, temporal, anterior/posterior cingulate, and precuneus regions of interest (ROIs) normalized to cerebellar-crus. Flortaucipir (AV-1451) PET provides information about tau tangle deposition. Flortaucipir PET images from 56 participants were used to assess meta-ROI [31] which was derived from uptake in entorhinal and inferior temporal ROIs. An automated pipeline based on Statistical Parametric Mapping (SPM12) platform [http://www.fil.ion.ucl.ac.uk/spm/] were used for PET analysis. All PET modalities were corrected for inter-frame motion, summed, and co-registered to matching T1 and spatially normalized to the MNI template space. Regional and composite PET indices were then derived as previously described [27, 30, 31]. PET scans were performed on a Siemens EXACT HR + or a GE Discovery PET/CT scanner at BAI. A 30-min (5-min × 6) dynamic emission scan is performed after intravenous (IV) administration of 5mCI of 18F-FDG, and a 30-min radiotracer uptake period. A 20-min (5-min × 4) PIB PET scan was performed following IV administration of 15 mCi of 11C-PIB and a 50-min uptake period, and a 18F-flortaucipir scan was acquired with IV injection of 10 mCi of the tracer with 75-min uptake time and 30-min (5-min × 6) dynamic scan. All PET images were reconstructed using an iterative algorithm with corrections of randoms, scattering, and attenuation correction using standard methods.

### MRI measures

Volumetric MRI measurements of hippocampal volume (HV) from 194 participants were corrected for intracranial volume were derived using FreeSurfer 6.0 using the default Desikan-Killian atlas [32]. Left and right hippocampal volumes were summed and normalized by total-intracranial-volume. Measures of cortical thickness for the entorhinal cortex, inferior temporal lobe, and parahippocampal regions were also analyzed. White matter hyperintensity volume (WMHV) corrected for intracranial volume (*n* = 150) was used to assess the degree of cerebrovascular damage and was quantified from T2-FLAIR images using the Lesion Growth Algorithm in the Lesion Segmentation Tool Box [33] in SPM12 with the detection threshold set to 0.15. A T1-weighted volumetric IRSPGR sequence (TE = Min Full, Flip Angle = 11, NEX = 1, FOV = 24, imaging matrix = 256 × 256, slice thickness = 1.2 mm) and a 3D T2-weighted FLAIR sequence were acquired during a single MR session on either a HR + 1.5 T (14% of sample) or a GE Discovery 750 3 T (86% of sample) scanner at Banner Alzheimer’s Institute.

### Statistical analysis

The Kruskal–Wallis test was used to assess unadjusted *APOE* genotype differences for age, education, neuroimaging, plasma NfL, and cognitive measures. Chi-square analysis was used to test for differences in sex frequencies across *APOE* genotypes. *APOE* ε4 gene-dose effects on plasma NfL were assessed using a generalized linear model (GLM) where a gamma distribution with a logarithmic link [34] was used to model the error of plasma NfL after adjusting for age, sex, and education. GLMs were also used to assess the associations between plasma NfL and neuroimaging measures after adjusting for age, sex, education, and *APOE* ε4 allelic dose. This particular statistical approach was used as the error distribution for plasma NfL did not meet the assumption normality due to its significant right skewness (Supplemental Fig. 1).

Delayed recall (DR) and Total Learning (TL) measures from the Rey Auditory Verbal Learning Test (AVLT) were used to assess the association between plasma NfL and episodic memory. Previous work by our group has shown that performance on these cognitive measures is differentially impacted by *APOE* ε4 gene-dose13-15. In addition, the association between plasma NfL and a cognitive composite score (AVLT Total Learning, AVLT Delayed Recall, Rey Complex Figure Copy, Rey Complex Figure Recall, WAIS-III Digit Span, WAIS-III Block Design, Judgment of Line Orientation, Controlled Oral Word Association Test, and Boston Naming Test) was also examined. Raw scores for each test were *z*-transformed using the mean and standard deviation of the sample, and the mean of the resulting *z*-scores was used as the composite measure.

False discovery rate (FDR) was used to corrected for multiple comparisons and all analyses were carried out using R 4.1.3.

## Results

**Fig. 2:**
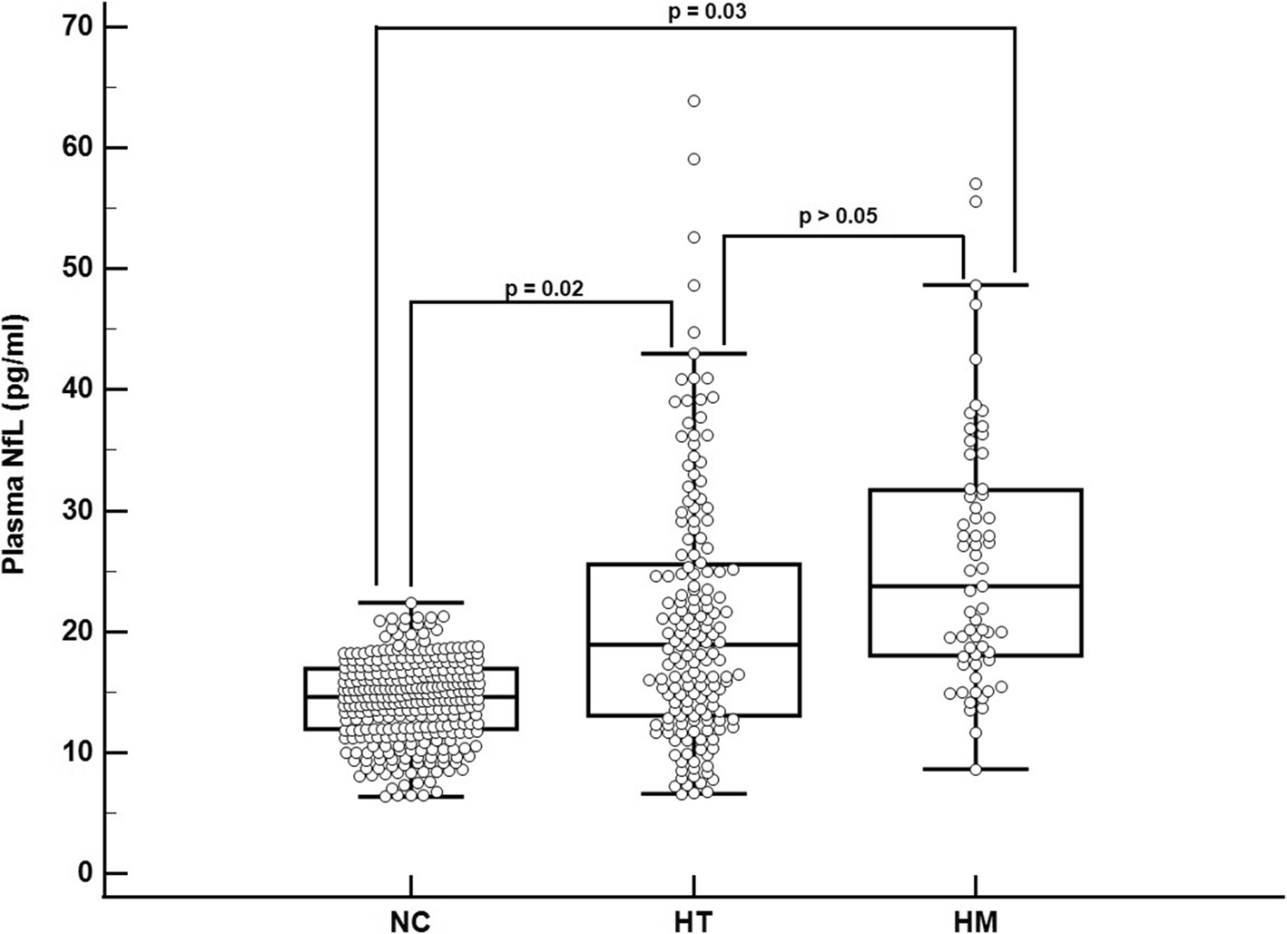
Adjusted plasma NfL levels in cognitively unimpaired *APOE* ε4 non-carriers (NC), heterozygotes (HT), and homozygotes (HM)

**Fig. 3:**
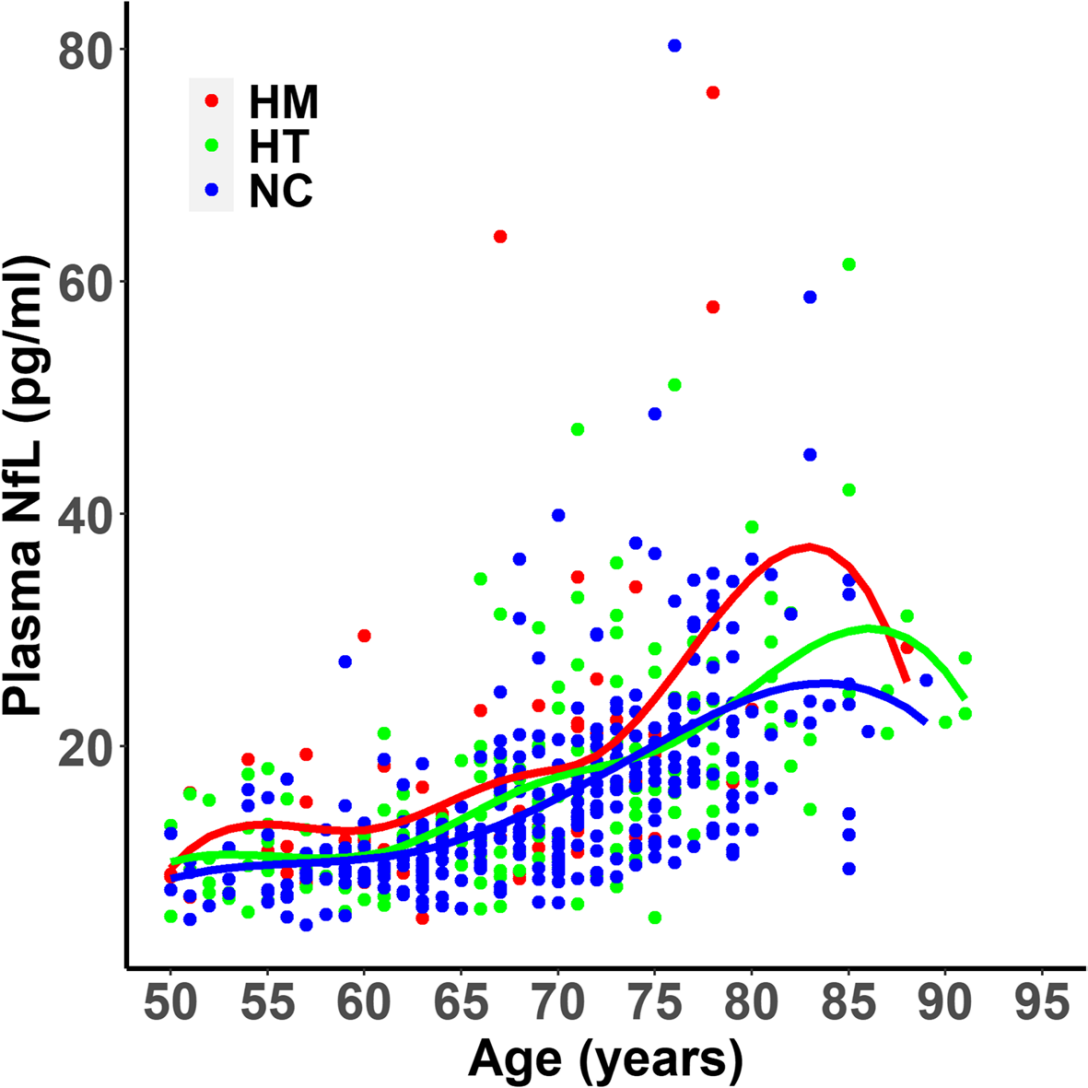
Age-associated plasma NfL changes stratified by *APOE* genotype

Descriptive data for demographic, plasma NfL, neuroimaging, and cognitive variables are shown in Table 1. Age (*p* = 0.10) and education (*p* = 0.91) were not significantly different between HMs, NTs, and NCs, and there was no significant difference in sex distribution among these groups (*p* = 0.75). Unadjusted plasma NfL was not significantly different between *APOE* genotypes (*p* = 0.46); however, after adjusting age, sex, and education both HMs (*p* = 0.03) and HTs (0.02) had significantly greater plasma NfL levels when compared to NCs (Fig. 2), while the difference between HTs and HMs was not significantly different (*p* > 0.05). Age-related associations with plasma NfL stratified by *APOE* genotype is shown in Fig. 3 where HMs have the greatest age-associated increases in plasma NfL relative to HTs and NCs whose plasma NfL levels begin to converge at age 70 with homozygotes showing a marked increase between ages 75 and 85. In general, plasma NfL appeared to increase linearly in the 50 to 70 age range, while a cubic pattern of increase was noted after age 70 for NCs, HTs, and HMs. An additional regression model tested the interaction between sex and *APOE* ε4 carrier status on plasma NfL and found that the interaction was not statistically significant (*p* = 0.47).

**Table 1:** Participant characteristics, plasma NfL levels, brain imaging measurements, and memory test scores

	**Homozygotes (HM)** ***n* = 66**	**Heterozygotes (HT)** ***n* = 165**	**Non-carriers (NC)** ***n* = 312**	***P*-value**	**Groupwise comparisons**
**Age (years)**	66.8 ± 8.3	68.8 ± 9.6	69.3 ± 8.1	0.10	na
**Education (years)**	16.0 ± 2.5	16.1 ± 2.1	16.1 ± 2.2	0.91	na
**Sex (M/F)**	16/50	46/119	90/222	0.75	na
Amyloid positive (%)	10% (6/61)	26% (8/31)	36% (8/22)	0.01	na
**MMSE**	29.1 ± 1.4	29.4 ± 0.9	29.6 ± 0.9	0.001	NC, HT > HM
**Plasma NfL**	18.0 ± 12.4	17.1 ± 9.1	16.2 ± 8.8	0.46	na
**Adjusted plasma NfL**	15.0 ± 3.5	15.5 ± 4.0	14.4 ± 3.4	0.005*	HM, HT > NC
**Hypometabolic convergence index**	9.9 ± 5.2	10.0 ± 5.4	7.7 ± 3.1	0.01	HT > NC
**Relative hippocampal volume**^**a**^	5.2 ± 6.8	5.2 ± 7.5	5.4 ± 6.3	0.10	na
**White matter hyperintensity volume (cm**^**3**^**)**	5.7 ± 9.3	5.2 ± 7.0	5.3 ± 7.7	0.98	na
**PiB SUVR**	1.4 ± 0.3	1.4 ± 0.3	1.2 ± 0.2	0.001	HM, HT > NC
**AV-1451 tau meta ROI**	1.2 ± 0.1	1.2 ± 0.2	1.2 ± 0.1	0.66	na
**AVLT total learning**	43.0 ± 10.9	44.9 ± 10.7	46.6 ± 10.5	0.03	HM < NC
**AVLT delayed recall**	7.3 ± 4.2	8.2 ± 3.9	8.7 ± 3.6	0.03	HM < NC

^a^Scaled to 1000X

For the neuroimaging variables, HCI was significantly greater in HTs relative to NCs (*p* = 0.01); however, HMs were not significantly different compared to the NC and HT groups. HMs and HTs had significantly greater PiB SUVR levels when compared to NCs (*p* = 0.001). HV, WMHV, and tau meta-ROI showed no significant *APOE* genotype differences (*p* = 0.10, *p* = 0.98, *p* = 0.66, respectively).

**Fig. 4:**
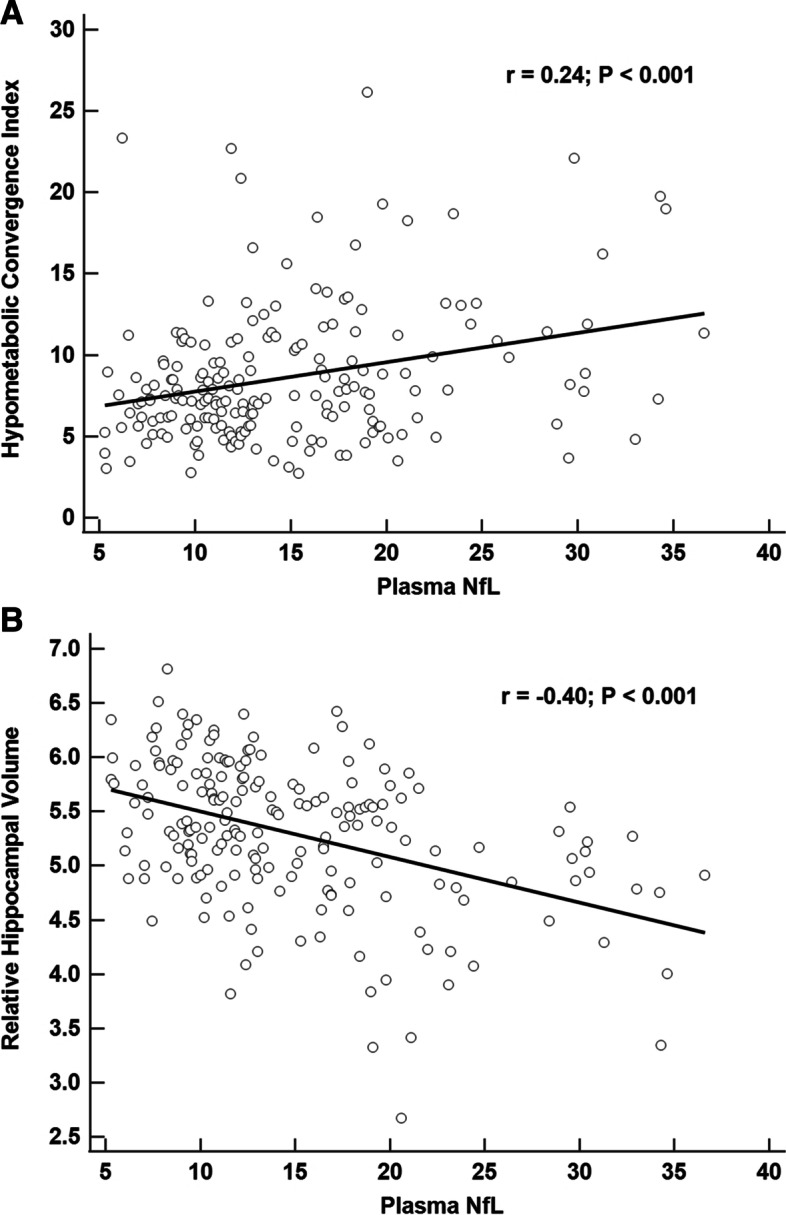
Plasma NfL associations with hypometabolic convergence index (**A**) and hippocampal volume (**B**)

Results for the GLMs that assessed plasma NfL and neuroimaging marker associations are shown in Table 2. After adjustments for age, sex, education, and *APOE* genotype the HCI (*β* = 0.01, 95% CI: (0.002, 0.03), *p* = 0.03; Fig. 4A) and HV (*β* =  − 0.12, 95% CI: − 0.21, − 0.02), *p* = 0.02; Fig. 4B) were significantly associated with plasma NfL; however, the HCI association did not survive multiple comparison adjustment. WMHV (*β* = 0.002, 95% CI: (− 0.006, 0.01), *p* = 0.56), PiB SUVR (*β* = 0.06, 95% CI: (− 0.31, 0.44), *p* = 0.75), and tau meta-ROI (*β* = 0.85, 95% CI: (− 0.02, 1.77), *p* = 0.07) were not associated with plasma NfL. PET measures of tangle burden in the entorhinal cortex and inferior temporal lobes showed significant unadjusted correlations with plasma NfL (*r* = 0.49, *r* = 0.52, respectively); however, only the entorhinal cortex association remained significant after covariate and multiple comparison adjustment (*β* = 0.95, 95% CI: (0.23, 1.68), *p* = 0.01). Thickness measures for entorhinal cortex (*β* =  − 0.10, 95% CI: (− 0.23, 0.02), *p* = 0.11) and inferior temporal (*β* =  − 0.10, 95% CI: (− 0.20, 0.007), *p* = 0.07) were not associated with plasma NfL; however, parahippocampal thickness was (*β* =  − 0.25, 95% CI: (− 0.42, − 0.08), *p* = 0.005). Sex interactions with WMHV (*p* = 0.74), entorhinal thickness (*p* = 0.60), inferior temporal thickness (*p* = 0.19), and parahippocampal thickness (*p* = 0.28) for plasma NfL were not statistically significant.

**Table 2:** Neuroimaging predictors of plasma NfL

Neuroimaging variable	Beta value	95% confidence interval	*P*-value	MSE
**Relative hippocampal volume (*n* = 192)**^a^	− 0.12	(− 0.21, − 0.02)	0.02	0.13
**Hypometabolic convergence index (*n* = 194)**	0.01	(0.002, 0.03)	0.03*	0.12
**White matter hyperintensity volume (*n* = 150)**	0.002	(− 0.006, 0.01)	0.56	0.12
**PiB SUVR (*n* = 110)**	0.06	(− 0.31, 0.44)	0.75	0.17
**Tau meta ROI (*n* = 56)**	0.85	(− 0.02, 1.77)	0.07	0.12
**Tau entorhinal SUVR (*n* = 56)**	0.95	(0.23, 1.68)	0.01	0.10
**Tau inferior temporal SUVR (*n* = 56)**	0.66	(− 0.08, 1.41)	0.09	0.12
**Entorhinal cortex thickness (*n* = 156)**	− 0.10	(− 0.23, 0.02)	0.11	0.14
**Inferior temporal thickness (*n* = 156)**	− 0.10	(− 0.20, 0.007)	0.07	0.14
**Parahippocampal thickness (*n* = 156)**	− 0.25	(− 0.42, − 0.08)	0.005	0.13

All models adjusted for age, sex, education, and *APOE* ε4 genotype; for PiB SUVR, only 19% of subjects were amyloid positive (SUVR >  = 1.47)

^a^Scaled to 1000X; MSE—mean squared error of regression model where values closer to zero indicate better fit. False discovery rate significance level was *ɑ* = 0.025

^*^Not statistically significant after FDR adjustment

**Fig. 5:**
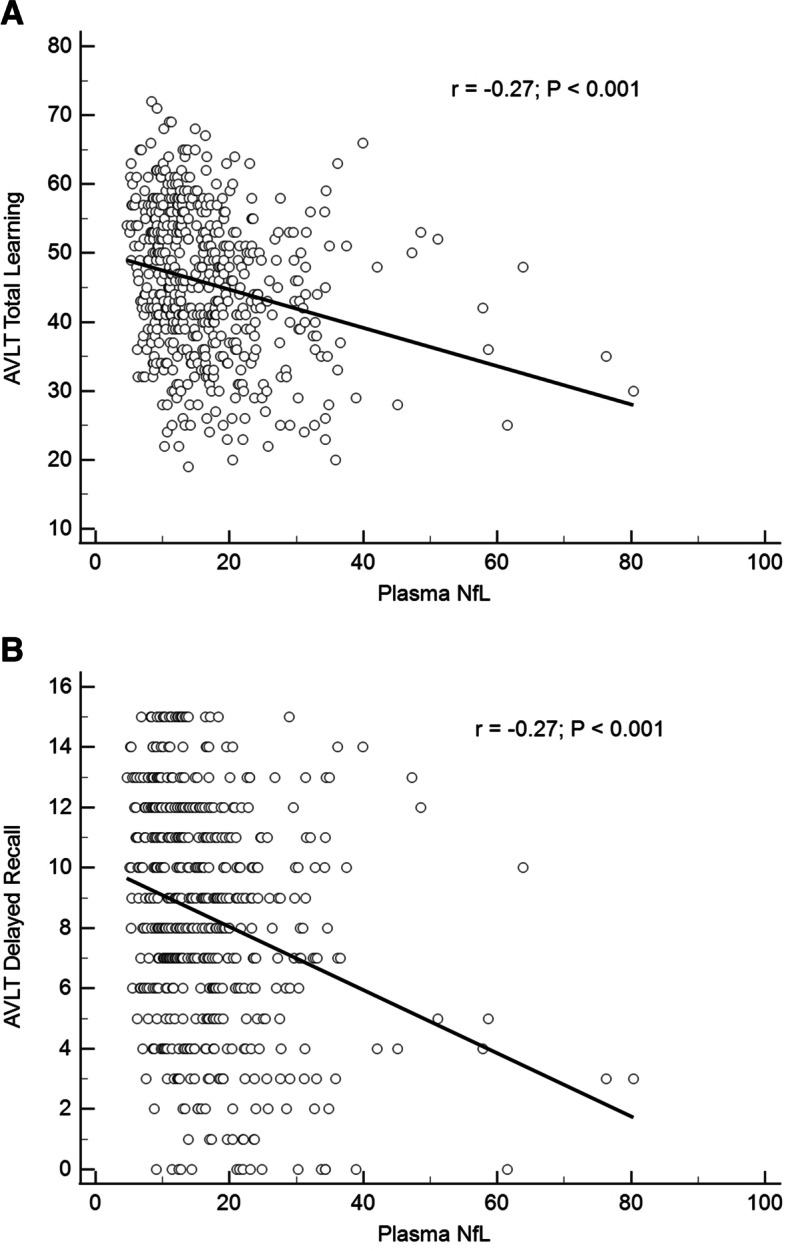
Plasma NfL correlations with AVLT total learning (**A**) and delayed recall (**B**)

For the cognitive variables, HMs had significantly lower AVLT-TL and AVLT-DR scores relative to NCs (both *p* = 0.03). Significant correlations were noted for plasma NfL with AVLT-TL (*r* =  − 0.27, *p* < 0.001; Fig. 5A) and AVLT-DR (*r* =  − 0.27, *p* < 0.001; Fig. 5B); however, these associations were no longer significant after demographic and *APOE* adjustment. The cognitive composite score also failed to show a significant association with plasma NfL after demographic and *APOE* adjustment (*β* =  − 0.02, 95% CI: (− 0.09, 0.05), *p* = 0.55). For comparison, the correlations with HV were *r* = 0.35, *p* < 0.001 for AVLT-TL and *r* = 0.38, *p* < 0.001 for AVLT-DR.

## Discussion

This study characterized plasma NfL measurements in cognitively unimpaired late-middle-aged and older adult *APOE* ε4 homozygotes, heterozygotes, and non-carriers. *APOE* ε4 homozygotes and heterozygotes had higher plasma NfL concentrations than non-carriers of this allele. As predicted, higher plasma NfL levels were significantly associated with MRI evidence of hippocampal atrophy, decreased parahippocampal thickness, and lower episodic memory scores, even before the onset of cognitive impairment. Higher plasma NfL also correlated with greater PET-based tangle load in the entorhinal cortex. The associations of plasma NfL with neuroimaging measures support its use as a BBBM of neurodegeneration as significant associations were noted for hippocampal volume, episodic memory performance, while CMRgl reductions in brain regions preferentially affected by AD [27] were statistically significant prior to multiple comparison adjustment. Previous studies have established that longitudinal declines in NfL correlate well with ATN status [35], AD-associated clinical changes [3], and faster rates of decline in cognition and cortical atrophy [36], so it is likely that plasma NfL can serve as a reliable marker for disease-modifying treatments in AD. The use of plasma NfL as a surrogate marker of cognition would also help avoid some of the methodological problems associated with cognitive outcomes such as practice effects, rater drift, and intrasubject variability [37]. However, the clinical utility of plasma NfL is uncertain as recent evidence suggests that it adds marginal diagnostic value when used with other clinical assessments [38] and has poor diagnostic accuracy for AD [39].

Findings from this study are consistent with other studies showing that higher plasma NfL levels are associated with hippocampal atrophy and CMRgl reductions in brain regions preferentially affected by AD [6], and they extend these relationships to CU individuals at increased genetic risk for late-onset AD. Others have shown that higher NfL levels are associated with PET measurements of Aβ plaque and tau tangle deposition [36]; however, these findings were in amyloid-positive individuals who were more likely to have amyloid-related tau pathology. The lack of association between plasma NfL and tau-PET meta-ROI in our data is surprising given this presumption; however, we did note that the tau-PET SUVR for the entorhinal cortex was significantly associated with plasma NfL which suggests that NfL and tau associations may be region-specific in the preclinical stages of AD.

The finding of higher NfL levels between *APOE* genotypes (HM, HT > NC) contrasts with recent reports that plasma NfL does not differ between *APOE* genotypes [40, 41]. The lack of difference in plasma NfL between *APOE* HTs and HMs we found could be due to the use of an asymptomatic cohort of individuals where plasma NfL changes may not be as pronounced. The demographically adjusted NfL values we found for HMs and HTs were nearly equal with standard deviations that far exceeded the difference in means indicating that assay variability may also obscure NfL differences between HMs and HTs.

There are some limitations to this study. For the Arizona *APOE* Cohort, not all individuals have amyloid and tau biomarkers so the extent to which the NfL changes are due primarily to preclinical AD is unclear. Moreover, the cohort is enriched for *APOE* ε4 allelic dose and is not a population-based study which does not allow for the discernment of relationships *APOE* ε4 HM, HT, and NC groups. The absence of random community-based sampling also introduces the risk of recruiting individuals concerned about their own cognitive status which might be due to early-stage AD in some. With regard to our findings, one potential weakness is that the plasma NfL association with cortical glucose metabolism was relatively small and did not survive multiple comparison adjustment despite our study utilizing a relatively large and well-characterized sample.

This study supports the use of plasma NfL assays to detect and track neurodegenerative changes and its potential to evaluate promising AD prevention therapies in cognitively unimpaired persons at genetic risk for late onset AD.

## Supplementary Information

**Additional file 1: Supplemental figure 1.** Distribution of Plasma NfL Values.

## Data Availability

Data used in this study can obtained by contacting Dr. Eric Reiman (eric.reiman@bannerhealth.com).
