# Supplementary material for: Plasma NfL is associated with the APOE ε4 allele, brain imaging measurements of neurodegeneration, and lower recall memory scores in cognitively unimpaired late-middle-aged and older adults
Source: Alzheimers Res Ther. 2023 Apr 10;15:74. doi: 10.1186/s13195-023-01221-w (PMC10084600; doi:10.1186/s13195-023-01221-w)
Supplement: Supplementary file 1 — Additional file 1: Supplemental figure 1. Distribution of Plasma NfL Values. [file 13195_2023_1221_MOESM1_ESM.docx]

Supplemental Figure 1. Distribution of Plasma NfL Values.


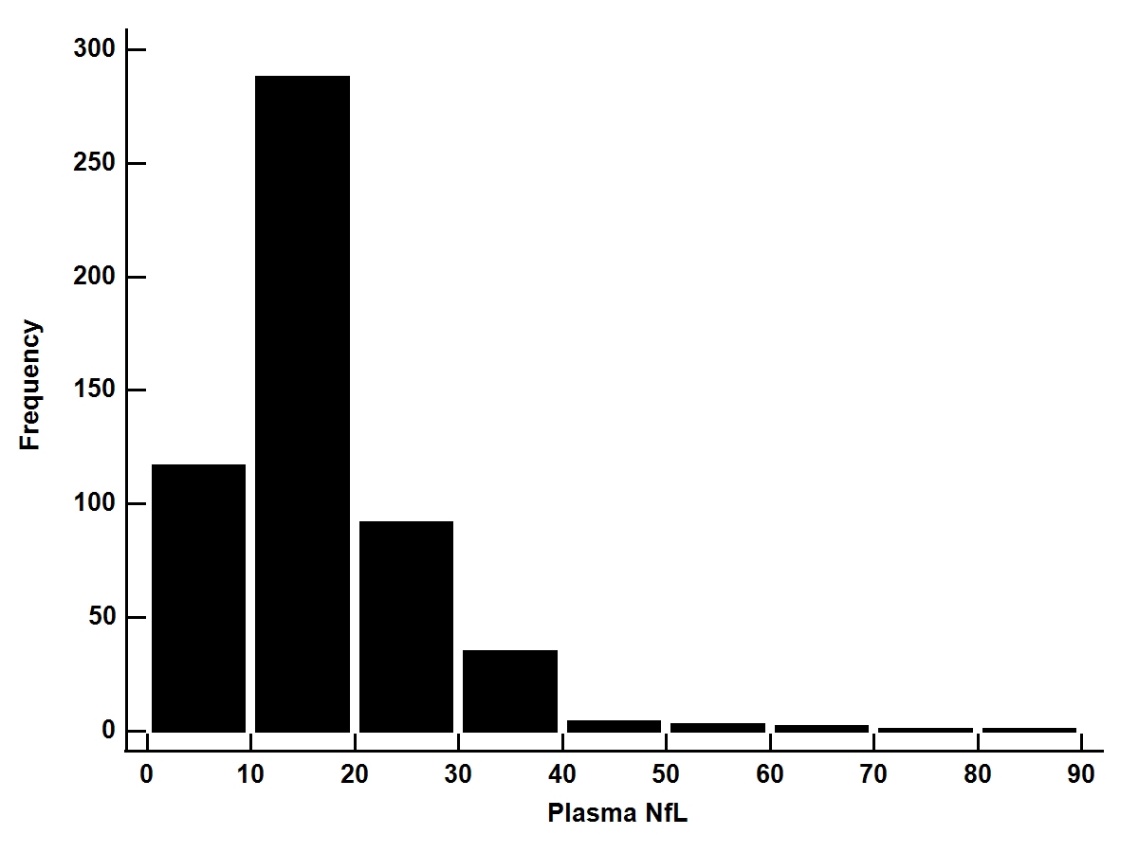


Supplemental Table 1. Neuroimaging Predictors of Plasma NfL Among APOE ε2/2 and ε2/3 Carriers.

| **Neuroimaging Variable** | **Beta Value** | **95% Confidence Interval** | **P-value** |
| --- | --- | --- | --- |
|  |  |  |  |
| **Relative Hippocampal Volume**† | -0.12 | (-0.52, 0.24) | 0.56 |
|  |  |  |  |
| **Hypometabolic Convergence Index** | 0.03 | (-0.03, 0.11) | 0.36 |
|  |  |  |  |
| **White Matter Hyperintensity Volume** | -0.004 | (-0.03, 0.02) | 0.73 |
|  |  |  |  |
| **PiB SUVR** | 0.85 | (-0.91, 2.64) | 0.45 |
|  |  |  |  |

All models adjusted for age, sex, and education; †scaled to 1,000X; None of the subjects in this analysis had flortaucipir data available.
